# Complete Heart Block Due to High Vagal Tone in Pregnancy

**DOI:** 10.1016/j.cjco.2025.06.001

**Published:** 2025-06-09

**Authors:** Ella Maria Cockburn, Jessica Yao, Robert Anderson

**Affiliations:** a Department of Cardiology, The Royal Melbourne Hospital, Parkville, Victoria, Australia; b Department of Cardiology, The University of Melbourne, Melbourne, Victoria, Australia

**Keywords:** pregnancy, complete heart block, vagal tone, atrioventricular block

**Atrioventricular (AV) block is rare in pregnancy.****^1^** **Most cases of complete heart block (CHB) that occur during pregnancy are due to congenital CHB that presents in the peripartum period. The pathogenesis of acquired CHB in pregnancy is not fully understood. Various mechanisms have been proposed, including atrial stretch and the effect of estrogen on estrogen receptors.****^2^** **We report a case of vagally mediated CHB. This condition causes variable intranodal AV block, which does not necessitate pacing and can be managed conservatively.**

## Case Presentation

A 32-year-old female gravida 3 para 0 patient presented at 32 weeks’ gestation with palpitations and exertional dyspnea without presyncope or syncope. Her past medical history was remarkable for an episode of Mobitz type I AV block 5 years prior. This episode was thought to have been precipitated by increased vagal tone in the setting of a precedent viral illness. She was managed conservatively at that time, with a return to sinus rhythm post–infection resolution. Other medical history included diet-controlled gestational diabetes. She was not on any AV nodal blocking agents or antiarrhythmic agents.

**Figure 1:**
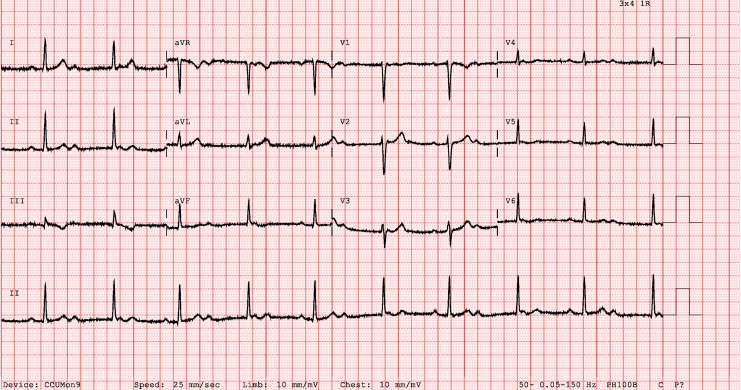
Electrocardiogram on hospital admission, demonstrating complete heart block with sinus nodal rate variability suggestive of increased vagal tone.

**Figure 2:**
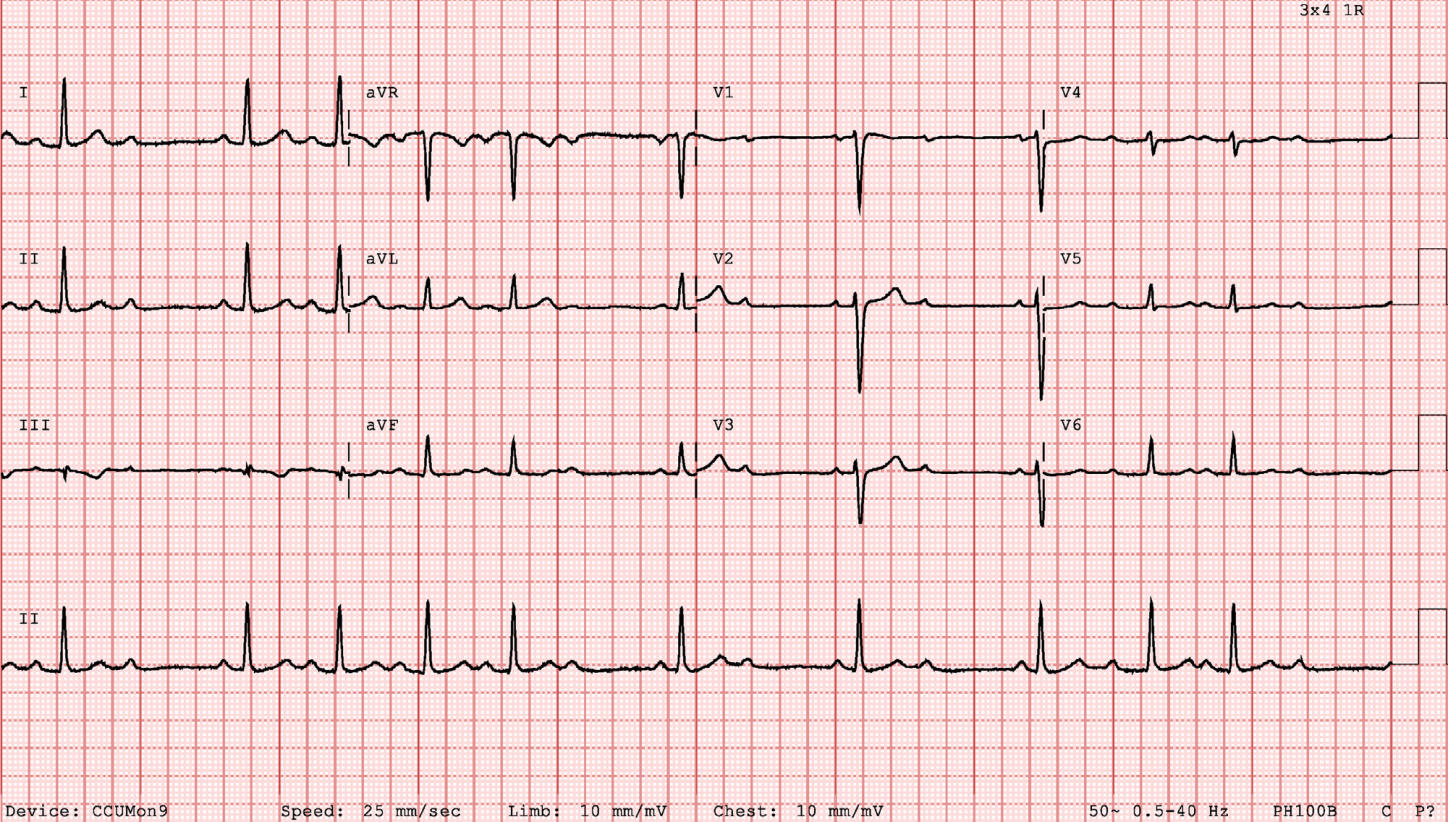
Electrocardiogram during hospital admission, demonstrating variable atrioventricular block.

Her cardiovascular examination was normal, and she was hemodynamically stable. Her white cell count was 10 x 10ˆ9/L (normal, 4.0-12.0 x 10ˆ9/L); her C-reactive protein level was < 5 mg/L, her troponin level was 2 ng/L (normal, < 11 ng/L), and her thyroid-stimulating hormone level was 0.92 mIU/L (normal, 0.13-4.55 mIU/L). Rheumatological screening was negative. An electrocardiogram (ECG) showed CHB (Fig. 1). Telemetry demonstrated intermittent 1st-degree AV block, Mobitz type I AV block, and CHB with a narrow escape rhythm (Fig. 2). A transthoracic echocardiogram demonstrated normal left ventricular systolic function, with no evidence of structural heart disease or valvular abnormalities. On an exercise stress ECG, her heart rate incremented to 115 beats per minute (61% maximum), with a peak blood pressure of 182/68 mm Hg. She had Mobitz type I AV conduction throughout the 7-minute test, which was limited by fatigue. A multidisciplinary meeting was held with the electrophysiologists and cardiac obstetrics team. The decision was made to discharge her to home with close monitoring. Subsequent outpatient ECGs demonstrated sinus rhythm.

A healthy baby girl was born at 38 weeks' gestation via emergency caesarean section with an epidural and sedation. This option was chosen due to a prolonged labour of 14 hours with poor cervical dilatation and subsequent fetal distress prompting delivery. The maternal rhythm was sinus throughout. The baby had a normal Apgar score and no conduction abnormalities. A postpartum maternal ECG demonstrated sinus rhythm with normal PR and QRS intervals. Subsequent cardiac magnetic resonance imaging demonstrated normal biventricular function and no myocardial edema or fibrosis. She has remained asymptomatic and in sinus rhythm since then.

## Discussion

Index presentations of acquired CHB in pregnancy are rare, with only 7 cases having been reported in the literature, of which only 2 cases did not receive a pacemaker.^2^^,^^3^ Several mechanisms have been postulated for acquired CHB in pregnancy, but these have not yet been proven correct. Increasing blood volumes associated with pregnancy may cause atrial stretch per the Frank-Starling law. This stretch may result in shortened refractoriness, slowed conduction, and spatial dispersion through activation of stretch-activated ion channels resulting in AV block. Another proposed mechanism is estrogen-mediated through the G-protein coupled estrogen receptor GPR30, which has a structure and function similar to those of beta adrenergic receptors.^2^

Another possible cause for CHB is increased vagal tone. Vagal tone is thought to increase throughout pregnancy due to aortocaval and diaphragmatic compression from the growing uterus.^3^ The vagal nerve depresses the sinus node and AV nodal conduction but does not influence the His-Purkinje system.^4^ Vagally mediated AV block is typically paroxysmal and recurrent; however, in states of overexcitation, such as pregnancy, this may lead to more persistent and recurrent AV conduction delay. The variability in the block seen in this case, from CHB to Mobitz type I and 1st-degree AV block, is in keeping with a vagally mediated block, vs an intrinsic AV block, which has less heterogeneity.^4^

Improvement in heart rate on exercise stress ECG testing can help differentiate between an intranodal vs an infranodal block. In this case, stress testing demonstrated heart rate incrementation, which is more in keeping with an intranodal block. The narrow QRS escape rhythm also is typical of an intranodal AV block. Intranodal block typically confers a better outcome than infranodal disease. Intranodal block usually presents with fewer symptoms, and has a better response to increased cardiac demand. By contrast, infranodal disease necessitates pacing.^5^ Monitoring in the postpartum period is paramount to determine whether a patient remains in CHB after delivery. Given the history of AV block, the reassuring laboratory and imaging results, and the resolution postpartum, this episode was presumed to be secondary to high vagal tone in pregnancy. If CHB had persisted postpartum, differentials would have included congenital CHB, myocarditis, infiltrative heart disease, infective endocarditis, and drugs.

CHB management in pregnancy typically is conservative if patients are hemodynamically stable and asymptomatic with a narrow junctional escape rhythm.^6^ Pacemaker implantation should be considered in pregnant patients if any of the following are present: syncope; prolonged pauses > 3x the cycle length of the escape rhythm; a prolonged QT interval; a mean daytime heart rate < 50 beats per minute; or a wide QRS interval.^6^ Fluoroscopy should be minimized in these cases due to risk to the fetus.

In this case, given the presumed vagally mediated mechanism and the relative clinical stability of the patient on discharge, close monitoring was performed with regular ECGs prior to delivery. A smartwatch or a long-term ECG event monitor could have been considered to allow for even closer surveillance. A clinician’s choice of rhythm surveillance strategy should take into account the symptom burden of the patient, the malignancy of their underlying rhythm, and the need for a strategy that allays, rather than provokes, health anxiety.

Neuraxial anesthesia can cause sympathetic blockade, and therefore should be avoided in patients with CHB. Accordingly, vaginal delivery is generally preferred.^2^ Fortunately, this patient underwent a successful emergency caesarean section without complications.

Further research is required to establish mechanisms of acquired CHB in pregnancy, but this case raises the possibility of a vagally mediated mechanism precipitating an intranodal block.

**Novel Teaching Points**

• An intranodal block can be differentiated from an infranodal block based on the QRS morphology, incrementation (or lack thereof) on stress testing, and variability in the degree of AV block seen.
• Distinguishing between intranodal and infranodal blockade is important, given that they have different management strategies.
• This case highlights the complexity of cardiac problems in pregnancy and emphasizes the importance of coordinating care among members of a multidisciplinary team, to optimize patient care.
